# Cross-Cultural Adaptation, Psychometric Properties and Measurement Invariance of the Italian Version of the Job Satisfaction Scale

**DOI:** 10.3390/ejihpe11030080

**Published:** 2021-09-16

**Authors:** Silvia Platania, Pasquale Caponnetto, Martina Morando, Marilena Maglia, Roberta Auditore, Giuseppe Santisi

**Affiliations:** 1 Section Psychology, Department of Educational Sciences, University of Catania, 95124 Catania, Italy; martina.morando@phd.unict.it (M.M.); gsantisi@unict.it (G.S.); 2 Center of Excellence for the Acceleration of Harm Reduction (COEHAR), University of Catania, 95124 Catania, Italy; pcapon@unict.it (P.C.); m.maglia@unict.it (M.M.); 3 Department of Educational Sciences, University of Catania, 95124 Catania, Italy; 4 CTA Psychiatric Rehabilitation and Research, 95030 Mascalucia, Italy; robauditore@gmail.com

**Keywords:** job satisfaction, psychometric properties, measurement invariance, Italian validation

## Abstract

The JSS is based on the theoretical position that job satisfaction represented an affective or attitudinal reaction to a job, and today is one of the most popular instruments used in I-O psychology. This paper discusses the contribution to the validation of an Italian adaptation of the Job Satisfaction Survey. Five hundred and twenty-seven participants (258 men, 269 women) were enrolled to participate in this study, aged between 19 and 65 (M_age_ = 36.0, SD = 11.7). The sample mostly worked in public administration, in health care, and in the educational sector. A self-report questionnaire is used to investigate the psychometric properties of this scale, also measuring other variables. A back-translation procedure is used. The results pinpointed the goodness of the scale and the normality distribution. Confirmative factor analyses and multigroup confirmative factor analyses were performed to verify the factorial structure of the scale. The results confirmed the same factorial structure of the original version, suggesting a nine higher-order factor structure. The results from the multigroup confirmatory factor analysis showed that this factor solution was invariant across gender (men vs. women) and found evidence for metric invariance, uniqueness invariance, and scalar and structural invariance. The findings confirmed the applicability in the Italian context.

## 1. Introduction

Inside the work environment it is important to consider factors that can positively or negatively affect the workers’ well-being. Therefore, it is important to observe and evaluate how emotions such as happiness, stress and anxiety play a role in job satisfaction. “Job satisfaction”, is a set of combinations of psychological, physiological, and environmental factors that lead the person to be genuinely satisfied with his/her work [1].

Every worker plays a fundamental role in every context where a profession is practiced. The profits of work organizations also depend to a large extent on job satisfaction and organizational commitment [2]. Employee job satisfaction increases motivation, performance, and reduces absenteeism and turnover [2,3,4].

The concept of job satisfaction for employees is also closely linked to factors of an external nature to the person or salaries received, benefits and recognitions, collaboration between colleagues, and general working conditions. A key role is also given to the style of leadership of employers and their presence within the work environment [5,6] and in order to achieve a good work result and at the same time achieve a satisfaction and loyalty of the employee, it is necessary to have good management of human resources [3,7,8].

Research conducted by Kumari and colleagues [9] on job satisfaction has shown what may or may not increase the concept of well-being or work malaise. The first factor is communication. A good communication climate promotes satisfaction both on a personal and professional level. Within their work environment, employees who are free to communicate may feel freer to express their opinions and produce better [10]. Another aspect to be highlighted within the working environment is that of safety; safety stems from the awareness that workers must achieve long-term objectives which also favor the concept of a sense of belonging [11].

The dispositional theory [12] reaffirms the importance of considering the innate characteristics that allow workers to have a certain degree of general satisfaction and specifically to work satisfaction. The concept of job satisfaction is seen as something that is not changing as it tends to remain stable over time beyond job change.

The Job Satisfaction Survey, developed by Paul Spector [13] is a valid multidimensional instrument. It is one of the most frequently used job satisfaction tools, and many studies about its psychometrical features have been conducted [14,15,16].

The main purpose of this research is to translate and examine the structure and to evaluate the factorial validity and the internal consistency of the Italian version of JSS. The results of the validity and reliability studies of an instrument assessing job satisfaction are of importance in order to promote psychological wellbeing at workplace.

## 2. The Measurement of Job Satisfaction

During the last 100 years, job satisfaction is a construct that has received considerable attention. Thousands of articles outline that definition and its use in the human resources sector [17,18].

Most recent reflections focused mainly on the job satisfaction of individual employees [19]; on the correlation between job satisfaction and organizational performance [20,21]; and on the impact that job satisfaction had on the workers’ well-being and how it could reduce the possibility of dismissal [22]. Furthermore, several factors considered as predictors have been associated with job satisfaction, as the nature of work, the job performance, and the type of organization [13,22]. The involvement, the commitment, and the interest that employers had in their work were also indicated as good antecedents [17,23,24].

Despite the well-defined and widely studied construct of job satisfaction, its measurability shows its limits. There have been many debates on how to measure this construct and how many are the predisposing factors. The current literature describes the job satisfaction construct both as a general criterion (e.g., job in general scale (JIG)) [25], neither as different dimensions and facets that can contribute to defined it, as well as communication, coworkers, fringe benefits, nature of work, organization itself, pay, promotion opportunities, recognition, security, and supervision (e.g., job diagnostic survey (JDS)) [26].

Thus, choosing the instrument that allow to measure the job satisfaction is a complex process. It is important to build methodological approach based on the hypotheses, the enrolled sample, and the research methodologies [27].

The large number of differences between the theory’s conceptualization and the related measures of job satisfaction could have a negative impact on researchers’ ability to carry out accurate and psychometric valid research in this field. Therefore, it seems necessary to provide a measurement scale that measures the construct in a coherent and accurate way regardless of the environment or the country. Moreover, it is important that the measuring instrument reports the same level of reliability and validity in a ‘wide variety of work contexts [28].

The Spector’s Job Satisfaction Survey could solve many of these problems related to measurement consistency since it is used in different work contexts with different samples of workers. For this reason, it is one of the most used job satisfaction measurement instruments validated in many countries around the world. The Job Satisfaction Survey investigates the job satisfaction in a generical way, but also it focuses on the different dimensions of this construct. For this reason, it is a multidimensional tool [29]. In addition, in 2003 a study conducted by Van Saane and colleagues [30] about the analysis on the psychometric validity of 29 job satisfaction measurement scales observed that the JSS was one of the seven tools that met the defined and validity criteria.

Job satisfaction is a fundamental element of employee well-being and the growth and improvement of organizations. In the Italian context, the JSS has not yet been validated. In order to adequately assess the attitudes and behaviours of employees towards their work, valid tools adapted in the local language are needed. Testing tools written in the local language and adequate culturally measure information in a more thoroughly way [31].

## 3. The Aim of Study

The Job Satisfaction Survey (JSS) was developed to fill the need for an instrument for human service, and public and nonprofit sector organizations (although it may be applicable to other contexts as well). Differing from other existing scales, it was developed to cover the main aspects of job satisfaction, with subscales clearly distinct in their content. The JSS was based on the theoretical position that job satisfaction represented an affective or attitudinal reaction to a job, and today is one of the most popular instruments used in I-O psychology.

This present study discusses the contribution to the validation of an Italian adaptation of the JSS, including evidence for reliability and validity. Specifically, it was examined: (1) the goodness of the scale and the normality of the distribution among the Italian and the American sample; (2) the fit indexes for models tested through confirmative factorial analysis; (3) the evaluation of the convergent and discriminant validity and the criterion validity of the scale; and (4) the description of the multi-group analysis used to investigate the differences for employment status.

## 4. Method

### 4.1. Participants and Procedure

The study involved a total of 527 participants (258 men, 49%; 269 women, 51%). The age of participants ranged between 19 and 65 (Mage = 36.0, SD = 11.7), 27.3 % working in public administration, 38.2% working in the health care sector and 34.5% working in the educational sector; hierarchical levels were bottom 46.5%, middle 43.6%, high 9.9%. With reference to the educational level, 30.8% of the sample completed 13 years of school, whereas the remaining 69.2% completed a minimum of 16 years of school.

A back-translation procedure is used for the adaptation to the Italian language, following the recommendations made by Beaton et al. [32]. The procedure followed these steps: translation and adaptation of the original scale from American to Italian, back translation, and a review committee. The first Italian version was then translated back to American by a bilingual psychologist with a doctoral degree. There was no substantial difference between the final Italian version and the original American one. The list of items in the original version and translated into Italian can be found in Appendix A.

The participants were enrolled through convenience sampling. Thanks to the collaboration with the company’s human resources department, the participants were involved in the study in two ways: 1) a link to the survey was published in the companies’ social media groups; and 2) via written correspondence (e.g., email or invitation by letter to participate). By clicking on the link, respondents were presented with a participant information sheet and an informed consent form, which, only once accepted, led to the survey with instructions on how to complete it. The participation was voluntary. Questionnaires were administrated individually and anonymously.

### 4.2. Measure

#### 4.2.1. Utrecht Work Engagement Scale (UWES)

The aim of this scale is to evaluate work involvement, meant as a positive and fulfilling state of mind related to personal work situation [33]. The Italian version of the scale is by Pisanti, Paplomatas, and Bertini [34]. Seventeen items form the scale, reflecting the three dimensions (vigor, dedication, and absorption) [35]. Responses to items were given on a frequency scale, using a Likert-scale varying from 0 (never) to 6 (always). In our study, the Cronbach alpha of the scale is 0.92

#### 4.2.2. Job Crafting Questionnaire (JCQ)

The scale was developed by Tims, Bakker, and Derks [36], and the Italian version is by Cenciotti et al. [37]. The aim of this scale is to measure job crafting, that is the set of proactive behaviours performed by employees to modify their working environment, adapting it to their needs and values. Fifteen items composed the questionnaire, investigating three positive dimensions: increasing structural job resources; increasing social job resources and increasing challenging job demands. Responses were given using a 7-point Likert scale, from 1 (never) to 7 (always), measuring the frequency with which one performs the different behaviours. In our study, the Cronbach alpha of the scale is 0.87

#### 4.2.3. Compound PsyCap Scale (Cpc-12)

The Compound Psychological Capital Scale (CPC-12), conceptualized by Lorenz et al. [38] (Italian version by Platania e Paolillo) [39], examines the psychological capital, defined as the pool of the personal positive resources, who affect the capability to achieve the desired goals [40]. The instrument consists of twelve items, containing the four components of psychological capital: optimism, resilience, hope and self-efficacy. For each statement, responses were given using a 7-point Likert scale ranging from 1 = strongly disagree to 7 = strongly agree. In our study, the Cronbach alpha of the scale is 0.93

#### 4.2.4. Job Satisfaction Survey

The 36-item Job Satisfaction Survey (JSS) [13] was used to measure job satisfaction. In the original English version of the Job Satisfaction Survey, nine subscales are included. Four items compounded each subscale, labelling: pay, promotion, supervision, fringe benefits, contingent rewards, operating conditions, co-workers, nature of work, and communication. The total score is computed from all items and subscales, using a 6-point Likert scale, from 1 = disagree very much to 6 = agree very much. Thus, the total score of job satisfaction ranging from 36 to 216. The scores of 4 to 12 represent dissatisfaction, 16 to 24 show satisfaction, and those between 12 and 16 represent ambivalence. For the total of items, the ranges are from 36 to 108 for dissatisfaction, from 144 to 216 for satisfaction, and between 108 and 144 for ambivalence. The JSS contains also self-descriptive positive and negative statements. The negatively worded items are reverse scored. Spector [29] reported a total scale Cronbach’s alpha coefficient from 0.91. to 0.75. This scale was translated into the Italian language using method of forward and backward translation by native speakers. For the improvement and assessment of these translations, we employed discussions with professionals, namely psychologists.

### 4.3. Data Analysis

The hypothesized model was verified using linear structural equation models. Tests were executed in AMOS 26.0, applying the maximum likelihood method to provide model parameter estimations simultaneously. As first step, several confirmatory factor analyses (CFA) were carried out on the dataset to detect the best factor model to fit the data. Moreover, different forms of equivalence were tested with series of multiple-group CFA, carried out on the whole sample grouped by gender [41,42]. Configural invariance, metric invariance [43], measurement error invariance [44,45], scalar invariance [46,47], and structural invariance [48,49] were tested.

The Tucker Lewis Index (TLI), the Comparative Fit Index (CFI), the Root Mean Square Error of Approximation (RMSEA), the GFI (Goodness Fit Index) and the Standardized Root Mean Square Residual (SRMR) were used to investigate the model’s goodness of fit. The values of RMSEA close to 0.06 were indicative of good fit, values between 0.07 and 0.08 are considered as moderate fit, and values between 0.08 and 0.10 were indicative of marginal fit. For the indexes CFI, GFI, and TLI, higher values proved better fit. The values above 0.95 pinpointed very good fit, values between 0.90 and 0.95 indicating marginally acceptable fit, and values lower than 0.90 showed poor fit.

χ2 values and ∆χ2 values were also used and were presented between the competing models, they assume multivariate normality and is sensitive to sample size. Owing to their sensibility to sample size [50], it was added the Akaike Information Criterion (AIC) and the Bayesian Information Criterion (BIC) (lower values indicate better fit). The index ∆CFI was also used with values not exceeding 0.01, indicating the equivalence of models in terms of fit [51]. It was used the Cronbach’s Alpha coefficient [52], to test reliability for the multiple-indicator construct, implemented by the measure of convergent validity testing with the average variance extracted (AVE) and the construct reliability (CR).

The AVE had to be values > 0.50 [53] and the CR had to be values > 0.60 [54]. Finally, to test the correlations, it was used SPSS 27.0.

To optimize the sample size, missing values for the relevant items were estimated using Expectation Maximization method. None of the items had more than 5 percent missing values, indicating that this option was appropriate for use [55].

## 5. Results

### 5.1. Descriptive Statistics and Normality of Distribution

In Table 1 it was reported the nine-facet scale, the overall means with the standard deviations of the Italian version compared to the American version and the normality of the distribution. The results confirmed the factorial structure of the scale, critical values that exceed +2.00 or that are smaller than −2.00 indicate statistically significant degrees of non-normality. Descriptive statistics in Table 2 showed that data were normally distributed, with acceptable skewness and kurtosis values. The results confirmed the goodness of the scale and the normality of the distribution.

### 5.2. Exploratory Factor Analysis

First, we conducted an exploratory factor analysis in order to verify the underlying theoretical structure of the and identify the underlying relationships between measured variables.

The Table 2 showed loading of the components and the communality of each item before rotation. Percent of total variance explained was 61.6%. Bartlett’s test of sphericity is significant (χ2 (630) = 8516.4, *p* < 0.001) and the test of Kaiser Meyer-Olkin (KMO = 0.92; BC = 0.849–0.956) it showed a good sampling adequacy of the data. As in the original version of Spector, the nine-factor structure is confirmed (Table 3).

In addition, the placement of the items in the factors are also confirmed: pay (Factor 1, items 1, 10, 19, 28), promotion (Factor 2, items 2, 11, 20, 33), supervision (Factor 3, items, 3, 12, 21, 30), fringe benefits (factor 4, item, 4, 13, 22, 29), contingent rewards (factor 5, items, 5, 14, 23, 32), operating conditions (factor 6, items, 6, 15, 24, 31), coworkers (factor 7, items, 7, 16, 25, 34), nature of work (factor 8, items, 8, 17, 27, 35) and communication (factor 9, items 9, 18, 26, 36).

### 5.3. Confirmatory Factor Analysis

As second step, a model with a first-order nine-factor structure and 36 second-order items (Model 1) was tested, and the following fit indexes were obtained: χ2(558) = 1946.62, SRMR = 0.058, RMSEA = 0.069, CFI = 0.92, TLI = 0.91,GFI = 0.91 AIC = 216.620, BIC = 262.477. Model 1 was then compared to a one first-order factor model and nine first-order factors (Model 2), [χ2(585) = 2239.28, SRMR = 0.067, RMSEA = 0.073, CFI = 0.80, GFI = 0.80, TLI = 0.78, AIC = 240.283, BIC = 276.926]. The first model of the two showed the best fit to the data, based on fit indexes, AIC, BIC and delta Chi-square value (Δχ²M2-M1(27) = 292.66). Owing to the close relationship between two of them Model 1 was then compared to a two-factor model, that might be more suitable than the nine-factor structure (Model 3). The findings expressed [χ2(594) = 3878.31, SRMR = 0.086, RMSEA = 0.103, CFI = 0.78, GFI = 0.77, TLI = 0.75, AIC = 422.311, BIC = 439.549] and it showed again the best fit to the data (Δχ²M3- M1(36) =1931.69). Moreover, all factor loadings were significant at *p* < 0.001 and varied between 0.641 and 0.839, with a mean of 0.73. Fit indexes for the tested models are presented in Table 4.

### 5.4. Discriminant Validity

Table 5 shows the intercorrelations, Cronbach’s α, composite reliability (CR) and average variance extracted (AVE) among nine facet scale of JSS. The results of Pearson’s bivariate correlation indicated a relationship between the low-medium variables, with a range between 0.12 and 0.48. These finding were similar to Spector’s results [13], which also reported low to medium intercorrelations among sub scales, ranging from 0.11 to 0.59. As Spector [29] reported, the JSS measured nine distinct aspects of job satisfaction, which was implied by discriminant validity, and for this reason they must had correlated moderation between them.

Cronbach’s Alpha was computed for each factor to test reliability and showed good internal consistency for the scale: pay 0.68, promotion 0.71, supervision 0.85, fringe benefits 0.69, contingent rewards 0.81, operating procedures 0.72, co-workers 0.71, nature of work 0.87 and communication 0.79.

Composite reliability and average variance extracted were CR 0.78, AVE 0.55 for pay, CR 0.84, AVE 0.60 for promotion, CR 0.81, AVE 0.57 for supervision, CR 0.79, AVE 0.58 for fringe benefits, CR 0.81, AVE 0.59 for contingent rewards, CR 0.75, AVE 0.52 for operating procedures, CR 0.76, AVE 0.54 for co-workers, CR 0.91, AVE 0.75 for nature of work and CR 0.84, AVE 0.60 for communication.

### 5.5. Criterion Validity

In this study, in addition to the discriminant validity, an analysis was carried out to test the criterion validity. This analysis was performed to verify the predictive validity of the nine sub scales of the JSS against other constructs widely present in the literature and related to job satisfaction.

Job crafting was defined as physical and cognitive changes in the tasks or relational restrictions of individuals [56] (p. 179). In many studies it was linked to job satisfaction because it is able to predict the level of job satisfaction of workers [57,58]. According to Yalabik and colleagues [59], work engagement had a high reliability in correlation with job satisfaction scales that used first of all a multidimensional approach. Finally, the literature reported the importance of a positive correlation between psychological capital and job satisfaction and how psychological capital is able to support the effects of job satisfaction in the long term [60,61,62].

The results showed in Table 6 confirm the predictive value of the variables, in particular the correlation between work engagement and promotion (r = 0.68, *p* < 0.001), between work engagement and fringe benefits (r = 0.60, *p* < 0.001), between job crafting and communication (r = 0.57, *p* < 0.001), between job crafting, and operating condition (r = 0.57, *p* < 0.001), between psychological capital and co-workers (r = 0.63, *p* < 0.001), and psychological capital and contingent rewards (r = 0.58, *p* < 0.001).

### 5.6. MCFA for Gender

According to Steyn and Bruin [63], differences in the results between groups (men vs women) may be depends of substantial differences between groups or may result from biased measurements (p. 2).

A multi-group analysis was tested on a configurational invariance model (Model 1), evaluating simultaneously the fit of male and female samples. The fit indexes of configural invariance (χ2(1116) = 2656.39, *p* < 0.001; CFI = 0.94; SRMR = 0.017; RMSEA = 0.051) indicated a good fit for this model and supporting an equivalent solution made of nine first-order factors for JSS in the data sets for both men and women (Table 7).

The fit of this configural model provided the baseline value against which all subsequently specified equivalence models are compared [48].

Model 2 was tested for metric invariance (Table 5). More importantly, Δχ2M2-M1(27) = 43.08 and ΔCFI = 0.000 suggested that Model 2 could be considered equivalent to Model 1. Thus, metric invariance was supported. Scalar invariance (Model 3) and error invariance (Model 4) they are also supported (Δχ2M3-M2(45) = 64.94, ΔCFI = 0.000; Δχ2M4-M3(36) = 88.39, ΔCFI = 0.000.

The equivalence in factor variances was tested (Model 5) and it was found to be coherent (Δχ2M5-M4(54) = 62.89, ΔCFI = 0.000). Finally, the equivalence in factor covariances was tested (Model 6) by nesting the respective model with Model 5, and the result was that it was supported (Δχ2M6-M5(23) = 96.41, ΔCFI = 0.000). The results were totally satisfactory as the model fit proved to be invariant across both populations (Table 5).

## 6. Discussion

Unlike other scales in the literature to measure job satisfaction, the Job Satisfaction Survey [13] presents nine different domains, each one capable of establishing a relationship or a specific cause that determines worker satisfaction.

The fundamental theoretical assumption of this scale is that job satisfaction represents an affective or attitudinal reaction to a job [13]. The attitudinal reaction implies positive and negative work behavior such as withdrawal towards work (e.g., turnover, absenteeism) or positive approach (e.g., high performance, motivation to work): these behaviors are related to job satisfaction. Thus, the JSS (Job Satisfaction Survey) is a multidimensional instrument, considering all these aspects.

The JSS is a widely used tool translated in more than nineteen languages.

In the context of organizational behavior studies, cross-cultural research is crucial to broaden the range of variations of the phenomena studied.

Nevertheless, cultural variability could influence the reading of a measurement scale. In some studies carried out in different countries to validate the psychometric properties of the instrument, it was demonstrated that cultural differences are the basis of the structure of the JSS [64].

Different factorial structures of the scale emerged in different countries, sometimes determining that cultural differences lie at the base of the scale structure [64] and sometimes confirming the original factorial structure, such as in Turkey and Pakistan [65].

In some cases, such as in Malaysia, Uganda, and Ukraine, the scale has been reduced to eight, four and three factors. In the most recent studies carried out in Greece and Croatia [66,67], on the other hand, the scale confirmed its goodness to nine factors and specifically, the Croatian study emphasized the first order structure of the scale with nine facet scales.

In all studies, however, the instrument appears to have good internal reliability and consistency.

The aim of this study was to examine the psychometric properties of the Job Satisfaction Scale in the Italian context.

Several quantitative procedures were used to confirm the psychometric properties in the Italian context. The confirmative factor analyses and the multigroup confirmative factor analyses using structural equation modeling (AMOS 26.0) were performed to verify the factorial structure of the scale. The results confirmed the same factorial structure of the original version [13], suggesting a nine higher-order factor structure.

Furthermore, it was carried out a multigroup confirmatory factor analysis to test whether the scale is invariant across gender. The measurement invariance was not assessed in the original study nor in the other validations present in literature in different cultural contexts. [13,66,67].

Measurement invariance was tested in the present study to progress the JSS validation, as only when such equivalence is established, researchers can proceed with examining mean group differences, having confidence that if any group differences are found, those are due to actual differences in psychological capital and not to an artefact of measurement error [68].

The findings from the multigroup confirmatory factor analysis showed that the same factor solution was invariant across gender (men vs. women). This means that Italians perceived the concept of job satisfaction and its nine components in the same way [48]. Furthermore, the present study found evidence for metric invariance, uniqueness invariance, scalar and structural invariance, which means that the relationship between the constructs was the same across the groups.

The reliability of the scale, evaluated by computing Cronbach’s alpha, composite reliability and average variance extracted (given the multidimensionality of the scale) showed good values.

Discriminant validity between latent factors [68,69] was tested using Fornell and Larcker technique [53], and it was fully supported.

Finally, criterion validity revealed significant high correlations between JSS and Job Crafting as well as Work Engagement and Psychological Capital, demonstrating that these variables were predictors of the investigated construct. All those finding supported the criterion validity of the scale and they advance the general claim of JSS to be applicable in Italian work context.

## 7. Conclusions

Compared to other countries where the Job Satisfaction Survey has undergone variations, in our study, on the other hand, the scale shows excellent adequacy in the Italian context.

We have conducted several statistical analyses that allow us to be able to state that the scale has good psychometric properties and is adaptable to measure job satisfaction within multiple work contexts.

The multidimensionality of the scale allows to verify different aspects of job satisfaction, this confirmation is important due to the various factors related to organizational well-being/malaise from which job satisfaction/dissatisfaction can arise [9,10] Unlike other pre-existing validations in others.

Differently to other pre-existing validations in other countries, the invariance by employment status has been included in our study, which verifies the versatility of the scale in all work contexts [14,15,16].

## 8. Limitations and Practical Implications

The study presents several psychometric solutions of the scale that until now had not been used. The results confirmed the validity of the scale within the Italian context. Although there were different measurement scales capable of measuring the construct of job satisfaction, the JSS has as an added value, due to the possibility of measuring the different attitudes that employees have towards work, considering its multi-dimensional aspects.

This is due to the fact that job satisfaction relates to all aspects of working life and favors push factors towards work such as self-efficacy [70] and prevents factors of distancing by favoring a good safety climate [71].

The study has some limitations that can be overcome in future research: data should be collected at different points in time, in order to allow verification of the predictive validity and test-retest reliability of the instrument; although statistical steps (Harman’s single-factor test) provided an indication that a single factor does not account for all co-variances among the items, it would be better to control for this effect at the research design stage.

Another very important limitation that we intend to overcome in future ongoing research is the fact that we used a single sample to carry out all the analyses. This choice was made due to the fact that we wanted to be consistent with the validations made so far in other countries. Finally, it would be necessary to compare studies from different countries with the same statistics to verify if there is a common denominator of interpretation of job satisfaction and its nine factors at the base.

## Figures and Tables

**Table 1:** Descriptive statistic, mean (M), standard deviation (SD), skewness and kurtosis.

	American Sample (N = 3067) Spector (1985)		Italian Sample (N = 527)			
	M	SD	M	SD	Skewness	Kurtosis
Pay	10.5	5.1	12.9	2.1	0.085	0.256
Promotion	11.5	5.1	10.9	2.8	0.217	−0.037
Supervision	19.9	4.6	11.6	1.9	0.085	0.497
Fringe_Benefits	13.1	5.0	12.5	2.0	0.095	0.849
Contingent rewards	13.4	5.1	13.1	2.8	−0.192	−0.752
Operating procedures	12.5	4.6	12.7	3.1	−0.209	−0.307
Co-workers	18.8	3.7	13.6	2.2	−0.116	0.420
Nature_of_work	19.2	4.4	14.3	2.2	−0.561	0.054
Communication	14.0	5.0	10.8	2.6	0.263	−0.241

**Table 2:** Exploratory factor analysis, unrotated loading matrix. Component loadings (N = 527).

	Factor 1	Factor 2	Factor 3	Factor 4	Factor 5	Factor 6	Factor 7	Factor 8	Factor 9	Communality
Item 1	−0.57	−0.35	0.07	−0.03	0.01	0.41	0.01	−0.10	0.17	0.68
Item 2	0.43	0.14	−0.29	0.24	0.10	0.48	0.21	0.16	−0.04	0.67
Item 3	−0.57	0.23	0.08	0.35	−0.05	−0.04	0.12	0.30	0.16	0.65
Item 4	0.55	0.24	−0.04	0.33	0.18	0.01	−0.02	0.06	−0.14	0.53
Item 5	−0.69	−0.02	0.12	0.21	0.07	0.04	0.20	0.05	−0.03	0.60
Item 6	0.22	0.06	0.43	0.18	−0.35	0.23	−0.06	−0.11	0.10	0.48
Item 7	−0.54	0.44	0.0	0.30	0.13	0.08	−0.32	−0.25	0.04	0.76
Item 8	0.47	−0.39	−0.018	0.51	0.18	0.10	−0.07	0.03	0.03	0.70
Item 9	−0.66	0.13	0.10	0.22	0.08	−0.01	0.08	−0.02	−0.13	0.56
Item 10	0.41	0.28	−0.29	0.10	0.09	0.42	0.23	0.01	−0.01	0.60
Item 11	−0.58	−0.28	0.27	0.09	0.14	−0.09	−0.02	0.10	−0.21	0.58
Item 12	0.59	−0.16	0.10	−0.16	0.35	−0.04	0.01	−0.22	−0.05	0.60
Item 13	−0.52	−0.31	0.210	0.13	0.03	0.27	0.14	−0.16	−0.149	0.58
Item 14	0.71	−0.02	0.10	0.14	0.31	0.01	−0.11	−0.01	−0.07	0.67
Item 15	−0.15	−0.03	−0.02	0.07	0.58	−0.14	0.43	−0.25	0.38	0.79
Item 16	0.48	−0.11	0.30	−0.15	−0.01	−0.04	0.38	0.13	0.08	0.54
Item 17	−0.40	0.53	0.24	−0.43	0.05	0.17	0.13	0.02	−0.12	0.76
Item 18	0.42	−0.20	0.23	−0.05	0.13	0.24	−0.21	0.43	−0.21	0.64
Item 19	0.67	0.27	0.16	0.15	0.18	−0.18	0.10	0.06	−0.26	0.74
Item 20	−0.45	−0.34	0.37	0.02	0.18	0.05	0.07	−0.05	−0.28	0.59
Item 21	0.65	−0.12	0.15	−0.16	0.09	0.23	−0.04	−0.39	−0.04	0.73
Item 22	−0.62	−0.17	−0.03	0.13	0.01	0.31	0.09	−0.07	−0.02	0.54
Item 23	0.68	0.38	−0.04	−0.09	0.13	0.05	−0.01	0.01	−0.02	0.64
Item 24	0.38	0.23	0.47	0.30	−0.28	−0.10	0.25	−0.18	−0.01	0.72
Item 25	−0.48	0.52	−0.02	0.25	0.18	0.02	−0.36	−0.23	0.03	0.79
Item 26	0.58	0.000	0.26	0.02	0.02	0.21	−0.25	0.13	0.13	0.55
Item 27	−0.43	0.53	0.25	−0.41	0.03	0.17	0.07	0.04	0.01	0.73
Item 28	−0.35	−0.06	0.44	−0.11	0.29	−0.10	−0.16	0.17	0.27	0.56
Item 29	0.56	0.35	0.20	0.01	0.13	−0.087	0.038	0.038	0.095	0.52
Item 30	−0.65	0.27	0.05	0.33	0.04	−0.085	0.039	0.319	0.142	0.75
Item 31	0.22	0.11	0.45	0.30	−0.43	−0.031	0.128	−0.147	−0.054	0.60
Item 32	0.75	0.29	0.19	0.02	0.05	−0.054	0.046	−0.048	−0.116	0.72
Item 33	−0.55	−0.16	0.48	0.08	0.20	−0.080	−0.097	0.074	−0.149	0.66
Item 34	0.56	−0.15	0.24	−0.21	−0.01	−0.004	0.064	0.155	0.175	0.50
Item 35	−0.61	0.44	0.13	−0.34	0.10	0.177	0.006	0.050	−0.060	0.76
Item 36	0.42	−0.09	0.36	0.02	−0.05	0.171	−0.177	0.042	0.422	0.56

**Table 3:** Exploratory factor analysis. Rotated loading matrix (N = 527) *.

	Factor 1	Factor 2	Factor 3	Factor 4	Factor 5	Factor 6	Factor 7	Factor 8	Factor 9
Item 1	−0.72	-	-	-	-	-	-	-	-
Item 2	-	-	-	-	-	-	-	-	-
Item 3	-	-	0.73	-	-	-	-	-	-
Item 4	-	-	-	0.34	-	-	-	-	-
Item 5	-	-	-	-	0.44	-	-	-	-
Item 6	-	-	-	-	-	0.61	-	-	-
Item 7	-	-	-	-	-	-	0.80	-	-
Item 8	-	-	-	-	-	-	-	−0.72	-
Item 9	-	-	-	-	-	-	-	-	0.41
Item 10	0.70	-	-	-	-	-	-	-	-
Item 11	-	0.63	-	-	-	-	-	-	-
Item 12	-	-	−0.58	-	-	-	-	-	-
Item 13	-	-	-	0.57	-	-	-	-	-
Item 14	-	-	-	-	−0.36	-	-	-	-
Item 15	-	-	-	-	-	0.86	-	-	-
Item 16	-	-	-	-	-	-	−0.59	-	-
Item 17	-	-	-	-	-	-	-	0.85	-
Item 18	-	-	-	-	-	-	-	-	0.43
Item 19	0.78	-	-	-	-	-	-	-	-
Item 20	-	0.72	-	-	-	-	-	-	-
Item 21	-	-	−0.74	-	-	-	-	-	-
Item 22	-	-	-	0.37	-	-	-	-	-
Item 23	-	-	-	-	−0.34	-	-	-	-
Item 24	-	-	-	-	-	0.76	-	-	-
Item 25	-	-	-	-	-	-	0.81	-	-
Item 26	-	-	-	-	-	-	-	-	0.55
Item 27	-	-	-	-	-	-	-	0.83	-
Item 28	0.43	-	-	-	-	-	-	-	-
Item 29	-	-	-	0.36	-	-	-	-	-
Item 30	-	-	0.76	-	-	-	-	-	-
Item 31	-	-	-	-	-	0.75	-	-	-
Item 32	-	-	-	-	0.65	-	-	-	-
Item 33	-	0.66	-	-	-	-	-	-	-
Item 34	-	-	-	-	-	-	−0.47	-	-
Item 35	-	-	-	-	-	-	-	0.76	-
Item 36	-	-	-	-	-	-	-	-	0.63

* Note: Loadings lower than 0.30 absolutes are omitted.

**Table 4:** Fit indexes for models tested in CFA.

	χ2	df	SRMR	RMSEA	RMSEA 90%-C.I.	CFI	TLI	GFI	AIC	BIC
Model 1 ^a^	1946.62	558	0.06	0.069	0.065–0.072	0.92	0.91	0.91	216.620	262.477
Model 2 ^b^	2239.28	585	0.07	0.073	0.070–0.077	0.80	0.78	0.80	240.283	276.926
Model 3 ^c^	3878.31	594	0.09	0.103	0.099–0.106	0.78	0.75	0.77	422.311	439.549

Note. ^a^ Model 1: nine first-order factors with co-variances among them; ^b^ Model 2: one second-order factor and nine first-order factors; ^c^ Model 3: all the items were predicted by a single factor; CFI = Comparative Fit Index; GFI = Goodness of Fit Index; TLI = Tucker Lewis Index; AIC = Akaike Information Criterion; RMSEA = Root Mean Square Error of Approximation; SRMR = Standardized Root Mean Square Residual; BIC = Bayesian Information Criterion.

**Table 5:** Reliability, composite reliability, average variance extracted, and intercorrelations (N = 527) *.

	α	CR	AVE	1	2	3	4	5	6	7	8
Pay	0.68	0.78	0.55	1							
Promotion	0.71	0.84	0.60	0.33 **	1						
Supervision	0.85	0.81	0.57	0.19 **	0.12 *	1					
Fringe Benefits	0.69	0.79	0.58	0.29 **	0.22 **	0.14 **	1				
Contingent rewards	0.77	0.81	0.59	0.30 **	0.36 **	0.25 **	0.20 **	1			
Operating procedures	0.72	0.75	0.52	0.12 **	−0.05	0.23 **	0.20 **	0.32 **	1		
Co-workers	0.71	0.76	0.54	0.16 **	−0.08	0.16 **	0.02	0.48 **	0.20 **	1	
Nature of work	0.87	0.91	0.75	0.13 **	0.21 **	0.15 **	0.12 **	−0.08	−0.023	0.12 **	1
Communication	0.79	0.84	0.60	0.20 **	0.10 *	0.16 **	0.10*	0.32 **	0.25 **	0.20 **	−0.022

Note: scores: * < 0.05, ** < 0.001.

**Table 6:** Correlations among variables *.

	Job Crafting	Work Engagement	Psycological Capital
Pay	0.54 **	0.55 **	0.38 **
Promotion	0.30 **	0.68 **	0.56 **
Supervision	0.51 **	0.45 **	0.48 **
Fringe Benefits	0.48 **	0.60 **	0.37 **
Contingent rewards	0.56 **	0.39 **	0.58 **
Operating conditions	0.57 **	0.45 **	0.55 **
Coworkers	0.46 **	0.47 **	0.63 **
Nature of work	0.34 **	0.35 **	0.41 **
Communication	0.57 **	0.50 **	0.55 **

Note: scores: * < 0.05, ** < 0.001.

**Table 7:** Fit statistics for measurement invariance by gender.

Model	χ2(df)	CFI	SRMR	RMSEA	ΔCFI
1. Configural Invariance	2656.39 (1116)	0.94	0.02	0.05 (0.048–0.053)	-
2. Metric Invariance	2700.19 (1143)	0.94	0.03	0.05 (0.048–0.053)	0.000
3. Scalar Invariance	2765.13 (1188)	0.94	0.03	0.05 (0.048–0.053)	0.000
4. Measurement error Invariance	2853.52 (1224)	0.94	0.03	0.05 (0.044–0.082)	0.000
5. Structural Variance Invariance	2916.41(1278)	0.94	0.03	0.05 (0.044–0.082)	0.000
6. Structural Covariance Invariance	3012.82 (1301)	0.94	0.04	0.05 (0.044–0.082)	0.000

## Data Availability

Not applicable.

## Appendix A

Item question: “Please circle the one number for each question that comes closest to reflecting your opinion about it” Response on a 6-point Likert scale: 1 (Disagree very much) to 6 (Agree very much)
1. Credo che mi paghino in modo adeguato per il lavoro che svolgo.
2. C’è ben poca possibilità che possa ricevere una promozione.
3. Il mio supervisore è molto competente nello svolgere il suo lavoro.
4. Non sono soddisfatto dei benefici che ricevo.
5. Quando svolgo un buon lavoro, mi vengono riconosciuti i miei meriti.
6. All’interno del mio lavoro vi sono molte regole e procedure che lo rendono difficoltoso.
7. Mi piacciono le persone con cui lavoro.
8. A volte ho la sensazione che il mio lavoro sia insignificante.
9. Considero di buon livello la comunicazione all’interno dell’azienda dove lavoro.
10. Non sono previsti aumenti di stipendio nel mio lavoro.
11. Coloro che svolgono bene il proprio lavoro hanno una buona possibilità di ottenere una promozione.
12. Il mio supervisore è ingiusto nei miei confronti.
13. I benefici che riceviamo sono buoni come quelli che vengono offerti nella maggior parte delle altre organizzazioni.
14. Ho la sensazione che il lavoro che svolgo non venga apprezzato.
15. I miei sforzi per fare un buon lavoro sono raramente ostacolati dalla burocrazia.
16. Credo che il mio lavoro sia reso più impegnativo a causa di alcune incompetenze di colleghi.
17. Mi piace fare le cose che faccio a lavoro.
18. Gli scopi di questa organizzazione non mi sono chiari.
19. Capita di non sentirmi apprezzato dall’azienda se penso al mio stipendio.
20. All’interno dell’azienda le carriere professionali si sviluppano velocemente come in altre realtà lavorative.
21. Il mio supervisore mostra poco interesse per i sentimenti dei subordinati.
22. L’insieme dei vantaggi che abbiamo sono equi.
23. Ci sono poche ricompense per coloro che lavorano qui.
24. Ho troppe cose da fare a lavoro.
25. Mi piacciono i miei colleghi.
26. Spesso ho la sensazione di non sapere che cosa stia succedendo in questa azienda.
27. Provo un senso di orgoglio nello svolgere il mio lavoro.
28. Mi sento soddisfatto della possibilità di un aumento del mio salario.
29. Esistono dei benefici di cui non possiamo usufruire ma che dovremmo avere.
30. Mi piace il mio supervisore.
31. Ho troppo lavoro da ufficio da svolgere
32. Non credo che i miei sforzi vengano premiati come dovrebbero.
33. Sono soddisfatto delle mie opportunità finalizzate a poter ottenere una promozione.
34. Ci sono troppi battibecchi e litigi sul posto di lavoro.
35. Il mio lavoro è piacevole.
36. I compiti da svolgere non sono spiegati in modo esaustivo.
